# Using behavioral insights to design implementation strategies in public mental health settings: a qualitative study of clinical decision-making

**DOI:** 10.1186/s43058-020-00105-6

**Published:** 2021-01-11

**Authors:** Briana S. Last, Simone H. Schriger, Carter E. Timon, Hannah E. Frank, Alison M. Buttenheim, Brittany N. Rudd, Sara Fernandez-Marcote, Carrie Comeau, Sosunmolu Shoyinka, Rinad S. Beidas

**Affiliations:** 1 Department of Psychology, University of Pennsylvania, Philadelphia, PA USA; 2 College of Liberal and Professional Studies, University of Pennsylvania, Philadelphia, PA USA; 3 Department of Psychology, Temple University, Philadelphia, PA USA; 4 Department of Psychiatry and Human Behavior, Warren Alpert Medical School of Brown University, Providence, RI USA; 5 Department of Family and Community Health, School of Nursing, University of Pennsylvania, Philadelphia, PA USA; 6 Center for Health Incentives and Behavioral Economics (CHIBE), University of Pennsylvania, Philadelphia, PA USA; 7 Penn Implementation Science Center at the Leonard Davis Institute of Health Economics (PISCE@LDI), University of Pennsylvania, Philadelphia, PA USA; 8 Department of Psychiatry, University of Pennsylvania Perelman School of Medicine, Philadelphia, PA USA; 9 Department of Psychiatry, University of Illinois at Chicago, Chicago, IL USA; 10 Community Behavioral Health, Philadelphia, PA USA; 11 Department of Behavioral Health and Intellectual Disability Services, Philadelphia, PA USA; 12 Department of Medical Ethics and Health Policy, University of Pennsylvania Perelman School of Medicine, Philadelphia, PA USA; 13 Department of Medicine, University of Pennsylvania Perelman School of Medicine, Philadelphia, PA USA

**Keywords:** Trauma-focused cognitive behavioral therapy (TF-CBT), Behavioral insights, Behavioral economics, Posttraumatic stress disorder, Implementation science, Implementation strategies

## Abstract

**Background:**

Trauma-focused cognitive behavioral therapy (TF-CBT) is an evidence-based intervention for youth with posttraumatic stress disorder. An important component of TF-CBT is the trauma narrative (TN), a phase in the intervention in which youth are guided to process the memories, thoughts, and feelings associated with their traumatic experience(s). Previous work has shown that TF-CBT clinicians complete TNs with only half of their clients, yet little is known about what determines TF-CBT clinicians’ use of TNs. The behavioral insights literature—an interdisciplinary field studying judgment and decision-making—offers theoretical and empirical tools to conceptualize what drives complex human behaviors and decisions. Drawing from the behavioral insights literature, the present study seeks to understand what determines clinician use of TNs and to generate strategies that target these determinants.

**Methods:**

Through semi-structured qualitative interviews, we sought the perspectives of trained TF-CBT clinicians working in public mental health settings across the city of Philadelphia (*N* = 17) to understand their decisions to use TNs with clients. We analyzed the qualitative data using a coding approach informed by the behavioral insights literature. We used an iterative process of structured hypothesis generation, aided by a behavioral insights guide, and rapid validation informed by behavioral insights to uncover the determinants of TN use. We then generated implementation strategies that targeted these determinants using the “Easy Attractive Social Timely” framework, a behavioral insights design approach.

**Results:**

We generated and validated three broad themes about what determines clinician implementation of TNs: decision complexity, clinician affective experience, and agency norms. We hypothesized behavioral insights that underlie these implementation determinants and designed a list of nine corresponding behavioral insights strategies that may facilitate TN implementation.

**Conclusions:**

Our study investigated why an effective component of an evidence-based intervention is difficult to implement. We leveraged robust scientific theories and empirical regularities from the behavioral insights literature to understand clinician perspectives on TN implementation. These factors were theoretically linked to implementation strategies. Our work revealed the potential for using behavioral insights in the diagnosis of evidence-based intervention determinants and the design of implementation strategies.

**Supplementary Information:**

The online version contains supplementary material available at 10.1186/s43058-020-00105-6.

Contributions to the literature

- This study identifies several determinants for why clinicians in public mental health settings complete the trauma narrative, a core component of trauma-focused cognitive behavioral therapy (TF-CBT), with only half of their clients.
- This work is unique in its integration of clinicians’ perspectives with the behavioral insights literature.
- This study illustrates a process for how to use behavioral insights—an interdisciplinary corpus of scientific theories and empirical findings—to understand clinicians’ decision-making and to design implementation strategies.
- These findings contribute to the work of TF-CBT implementation by unveiling the various ways clinicians in public, resource-scarce mental health settings face decision-making challenges.

## Background

There are numerous challenges to the implementation of mental health evidence-based interventions (EBIs; interventions supported by scientific evidence). These challenges are often specific to the structural and organizational factors that constitute the ecosystem of mental health service delivery, the individual decision-makers involved in implementation, and the client population [1–9]. Though these challenges are generalizable to the implementation of most mental health EBIs, there are also unique challenges to the implementation of specific mental health EBIs that require attention in the development and selection of implementation strategies.

First, mental health EBIs are complex and multicomponent. Most mental health interventions are designed and tested in efficacy trials as complete packages or manualized protocols, yet a limited set of core techniques and principles in mental health EBIs are responsible for their effectiveness [10–12]. With implementation in mind, there is growing recognition by researchers that it is essential to understand which EBI components account for therapeutic change [13, 14]. Once identified, implementation researchers can prioritize the specific components that have garnered the strongest evidence to target impediments to implementation and to design strategies to overcome these barriers. It is particularly important to target these components because these core techniques are often the most likely to be underused by clinicians [15–18].

Second, it is not always clear how to generate implementation strategies based on stakeholders’ first-person perspectives, even with the use of scientific frameworks. Implementation science has long recognized that qualitative research is essential to provide a textured understanding of clinicians’ experiences of EBI use [19, 20]. Thus, methods to design and select implementation strategies rely on stakeholder’s self-reported barriers and facilitators [21]. One challenge for EBI implementation is to reconcile the growing literature that shows that clinicians, like all humans, lack complete insight into their motivations and behaviors and, further, that their self-reports are conditioned by their organizational and broader social contexts [22]. One potential reconciliation is to apply scientific theories on judgment and decision-making to the analysis of stakeholder perspectives. That is, qualitative data can be leveraged to go beyond literal interpretation of clinician self-report—these data can be interpreted using scientific theories of the implicit processes that underlie judgment and decision-making to generate falsifiable causal hypotheses for why EBIs are challenging to implement and why strategies do or do not work.

### Behavioral insights

“Behavioral insights” (an umbrella term referring to discoveries from behavioral economics, cognitive science, and social psychology) can offer scientists tools to address the challenges of implementing complex EBIs [23]. Behavioral insights comprise a set of theoretical principles, frameworks, empirical regularities, and strategies derived from a decades-long, multidisciplinary effort to understand human judgment and decision-making [24]. These insights reveal the ways in which individuals make decisions—individuals tend to have incomplete information, work with enormous constraints on their time and resources, and employ heuristics, or mental shortcuts, to make decisions [25]. Behavioral insights demonstrate how people’s computational limits and motivated reasoning shape judgment and decision-making. Importantly, people are largely unaware of the biases and mental shortcuts they employ to make decisions [26]. This has implications for the interpretation of self-report data. If people are unaware of their motivations, judgments, and decisions, then self-report data on EBI implementation may benefit from interpretation using behavioral insights.

In addition to elucidating human judgment and decision-making, the multidisciplinary field of behavioral insights has generated strategies to improve decision-making. Rather than attempting to change the ways in which people are systematically biased, behavioral insights strategies leverage these systematic biases to optimize decision-making. A subset of these strategies, known as nudges, alter the “choice architecture,” or the way options are presented, to lead decision-makers to behave in predictable ways [27]. Nudges shape choice architectures to influence discrete, one-time decisions. For example, people overwhelmingly tend to choose the status quo or default option [28]. Nudges that make the optimal or more evidence-based decision the default have been effective across a host of domains including dietary choices, medical decisions, financial savings, and education [29–31]. Behavioral insights also encompass strategies that, unlike nudges, require sustained effort. For example, psychologists and implementation scientists have long-recognized that social motivation, incentives, and rewards are crucial levers of behavior change [32–37]. Studies from across the globe suggest that providing women with continuous social support during childbirth using a labor companion encourages them to be mobile, per guidelines, and improves health outcomes for women and babies [38]. Internationally, health service researchers and public health organizations are beginning to study behavioral insights to improve healthcare [39–48], and a new review calls for their integration into implementation science [49].

### Study context

This study was conducted in the city of Philadelphia, where the majority of treatment-seeking youth (55–80%) in the city receive public mental health services [50, 51]. Public mental health services, funded by Medicaid, are administered by the Department of Behavioral Health and Intellectual Disability Services (DBHIDS). Due to the high incidence of trauma exposure in Philadelphia, DBHIDS initiated a full-scale effort to develop a trauma-informed behavioral health system in 2011. In 2012, DBHIDS was awarded a National Child Traumatic Stress Initiative Community Treatment and Service Center grant (Category III) from the Substance Abuse and Mental Health Services Administration (SAMHSA) to form the Philadelphia Alliance for Child Trauma Services (PACTS). These grants support building the enduring infrastructure necessary to facilitate implementation rather than focusing on increasing the uptake of particular interventions [1]. PACTS represents a public-academic partnership that includes policy-makers, public mental health agency leadership, and university-based researchers who have worked collaboratively for the past decade to create a network of trauma-informed care in Philadelphia. In addition to increasing trauma screening and assessment and developing a robust crisis response service, PACTS has supported the training of clinicians in evidence-based trauma treatments.

Of these treatments, trauma-focused cognitive behavioral therapy (TF-CBT) has been a focus [50]. Over twenty randomized controlled trials (RCTs) show that TF-CBT is effective for youth with posttraumatic stress disorder [52–54]. Despite its research base, TF-CBT is not regularly implemented in public mental health settings [55]. Since 2012, ten cohorts of clinicians have been trained in TF-CBT across outpatient public mental health and residential treatment agencies through the PACTS initiative. Training includes 2 days of didactics followed by ongoing consultation provided via bi-weekly consultation calls for eight months with a TF-CBT certified master trainer. Throughout the year, PACTS-trained clinicians are offered to participate in “booster sessions” to fine-tune skills and seek clinical guidance. See [50] for more details on PACTS and TF-CBT training.

Dismantling research demonstrates that TF-CBT is more effective when the trauma narrative (TN) is used [56]. In the TN phase of treatment, the clinician guides the youth in sharing their memories, thoughts, and feelings related to the traumatic event. The narrative serves several purposes, including systematically desensitizing the child to traumatic memories as well as facilitating emotional processing of the memories to provide the child with a sense of mastery over their traumatic experiences. TF-CBT national trainers (i.e., expert TF-CBT clinicians who have trained over 5000 TF-CBT providers) have identified TN implementation as the most significant challenge to TF-CBT fidelity [17]. They speculated that clinician discomfort with a directive approach, fear of causing harm, and limited therapeutic skills beyond TF-CBT were significant barriers to TN implementation. To our knowledge, studies of clinicians’ perspectives of implementing TNs have not yet been published. However, TF-CBT clinicians in Philadelphia report completing TNs with only half of their clients [50].

### Objective

The present work examines perspectives from clinicians participating in city-wide implementation efforts in Philadelphia to (1) understand the implementation of an active yet underused component (the TN) of an effective and complex psychological EBI (TF-CBT) and (2) use principles from the behavioral insights literature to theoretically link this understanding to the development of implementation strategies.

## Methods

In 2018, we conducted qualitative interviews with PACTS clinicians across Philadelphia and asked about their decision-making processes implementing TNs. We adapted a behavioral insights approach to systematically stage the analysis—Narrow, Understand, Discover, Generate, and Evaluate (NUDGE)—and coded the interview data using a guide from the behavioral insights literature—the Behavioral Economics Guide—to arrive at behaviorally informed hypotheses about the determinants of clinicians’ TN use [57, 58]. We leveraged these hypotheses to generate implementation strategies using the behavioral insights-informed Easy Attractive Social and Timely (EAST) framework, which organizes strategies (both nudge and non-nudge) for researchers and policy makers [59].

### Participants and study procedure

Participants were clinicians who had completed training in TF-CBT through PACTS. Clinicians were contacted (either by e-mail or at a “booster” training session) in the spring of 2018 and asked to complete a 10–15-min survey about their perceptions of and past use of TNs. See [60] for more information about the initial survey clinicians completed. Of the 65 clinicians that completed the survey, a subset (*n* = 26) was selected for in-depth qualitative interviews using purposive sampling. Participants who completed qualitative interviews were sampled to capture variability in clinician TN use. On the survey, participants indicated the percentage of TF-CBT clients with whom they used TNs in the past 6 months, whether they intended to use TNs with their TF-CBT clients in the next six months, and how likely it was that they would use TNs with their TF-CBT clients in the next 6 months. Based on these responses, clinicians fell into three groups and were purposely sampled for qualitative interviews from each group, including (1) clinicians with high intentions and high likelihood of using TNs, but who had used TNs with none or few clients in the past (*n* = 8); (2) clinicians with high intentions and high likelihood of using TNs who reported using TNs with all or most of the their clients in the past (*n* = 5); and (3) clinicians who reported low intentions but medium to high likelihood of using TNs who had variable levels of past TN use (*n* = 4). Of the 26 participants who completed the survey and were invited to partake in the qualitative interviews, 17 (65%) participants completed interviews by phone or in person. Those who declined either did not respond to attempts to contact them or reported insufficient time to complete an interview. All procedures were approved by the City of Philadelphia and University of Pennsylvania Institutional Review Boards.

Semi-structured interviews focused on clinician perceptions of TNs, as well as factors that interfere with or assist their use. Several questions prompted clinicians to consider their most recent session with a client and the determinants to TN implementation in a single session [61]. These questions elicited concrete descriptions of clinicians’ judgment and decision-making in order to analyze the interviews using behavioral insights (see Additional file 1 for the interview guide).

Each participant completed one interview lasting between 30 and 60 min. The interviews were audio-recorded and conducted individually in person or by phone. BSL and HEF, both doctoral students familiar with TF-CBT and PACTS, conducted the interviews. Undergraduate research assistants transcribed the interviews. Participants received a $50 gift card.

### Analytic approach

We used an integrative approach informed by thematic analysis and a flexible adaptation of existing frameworks from the behavioral insights literature to interpret and code the qualitative data. As no single approach was sufficient to guide the hypothesis generation process, our study team integrated several guides and frameworks from the behavioral insights literature. Our analytic approach had three major phases, elaborated below.

First, in order to distill qualitative interview transcripts, thematic analysis was applied to organize the qualitative data into a manageable and interpretable amount of text [62]. Second, in order to systematize the hypothesis generation process, we selectively borrowed elements from the NUDGE framework, which has been used to design behavioral insights-derived implementation strategies based on hypothesized determinants [57]. To structure this phase, we relied heavily on the Behavioral Economics Guide to code hypothesized behavioral insights determinants of TN implementation [58]. Third, we used EAST to design behavioral insights-informed implementation strategies [59].

NUDGE is a behavioral insights approach that rigorously identifies what drives EBI implementation [57]. NUDGE lays out a multi-step process from “Narrowing” the focus to a specific behavioral target through “Understanding” the context of the behavior, “Discovering” the underlying behavioral insights, “Generating” implementation strategies, and “Evaluating” them through trials. In previous work, the NUDGE approach was used to analyze qualitative data to discover what drives EBI implementation in publicly-funded mental health agencies [57]. We adapted the “Discover” step of NUDGE into a coding process in which we applied codes for various behavioral insights largely drawn from the Behavioral Economics Guide 2018 [58]. Note that this guide is not exhaustive, and that given their training, coders were also familiar with other behavioral insights guides that they drew upon in this step [63]. To structure the “Generate” step of NUDGE, we used the EAST framework to propose behavioral insights-derived implementation strategies [59]. EAST was developed by the UK Behavioral Insights Team, a group of scientists and policymakers who apply findings from social psychology, cognitive science, and behavioral economics to a host of policy domains. EAST was developed as a practical and comprehensive tool for researchers and practitioners to arrange evidence in a digestible format. EAST primarily organizes behavioral insights strategies according to the principles that underlie their effectiveness. These strategies work because they make the optimal choice easier, more attractive, more social, and/or timelier than other choices. EAST offers a structured way to comprehensively consider all the mechanisms by which to address hypothesized implementation determinants.

It is important to note that, in the current study, we did not generate an exhaustive list of all potential implementation strategies. Rather, we designed several possible strategies to illustrate the promise of this structured brainstorming process.

#### Behavioral insights coding process

**Fig. 1:**
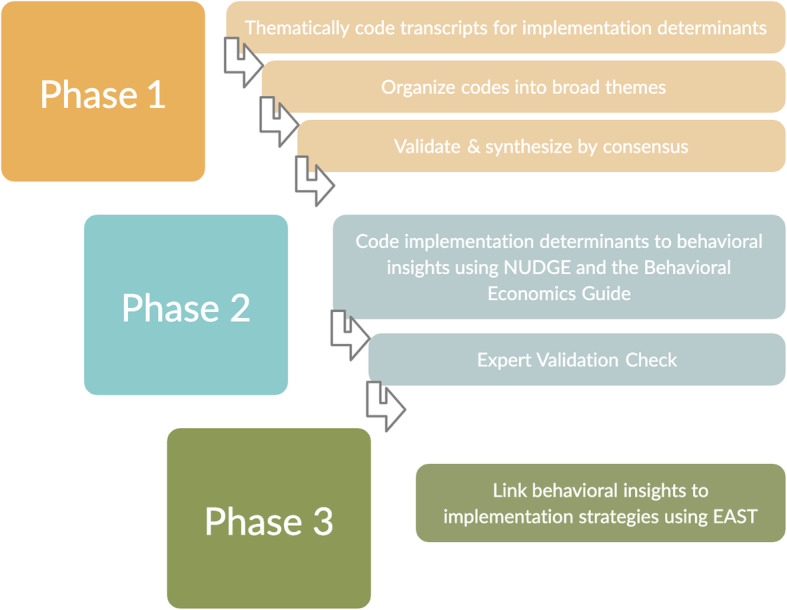
Steps of the qualitative analysis process*. Note*. See Table 3 for details on the final themes, TN determinants, behavioral insights, and implementation strategies developed out of this process

Figure 1 displays the multi-step process we used to analyze the qualitative interviews. The first phase of the coding process, described above, in which interviews were coded using thematic analysis, was conducted in Steps 1–3. The second phase of analysis, where we selectively borrowed elements from the NUDGE framework to iteratively map TN determinants onto behavioral insights using the Behavioral Economics Guide 2018 [58], was conducted in Steps 4–5. Table 1 provides definitions of the behavioral insights that we mapped onto the TN determinants. The third phase of analysis, in which we used EAST to design implementation strategies, was conducted in Step 6 [59]. See Additional file 2 for a full description of the coding process.

**Table 1:** Behavioral insights identified through coding process

Behavioral insights	Definition	How behavioral insight can determine TN implementation
Base Rate Fallacy/Mental Models	Base rate fallacy refers to when individuals ignore probabilities when making decisions and instead use the similarities between events to make predictions. Mental models are internal representations of the world.	Clinicians who experience the base rate fallacy may believe that aggregated data from efficacy trials, which are used to develop clinical practice guidelines, do not apply to their individual clients because of the perceived dissimilarity between their clients and trial participants. TF-CBT clinicians may have mental models of a “straight forward” or “typical” TF-CBT case, whereas other clients may align less with their image of the model of a typical TF-CBT case.
Choice Overload/Decision Fatigue	Choice overload occurs when decision-makers are faced with too many choices—the more choices, the more likely decision-makers will employ heuristics in lieu of reason. This relates to decision fatigue, or when people become fatigued the more decisions they make, which leads to poorer decisions.	Clinicians may feel that they don’t know how to choose among the many different intervention options (i.e., modality of the narrative, how to structure the narrative, etc.) they have at their disposal for a given client. They may feel psychologically taxed by the multiple decisions.
Default Bias	Default bias is the tendency for decision-makers to prefer the current state of affairs and an aversion to change.	Clinicians may prefer the current practices they implement in their clinical work. This occurs because the current treatments they are implementing are taken as a reference point, and any change from that baseline is perceived as less preferable.
Fear Avoidance/Ostrich Effect	Fear avoidance is the tendency to avoid thoughts or actions that cause people fear. The ostrich effect is related to fear avoidance; it describes people’s tendency to ignore or fail to seek, often negative, information.	Clinicians may avoid implementing the trauma narrative because it is difficult for them—they may not be as skilled in the trauma narrative as the practices they have been trained in, and therefore do not want to engage in something that makes them feel less competent or nervous. They may also fear the difficulty in hearing details they may learn during the trauma narrative.
Functional Fixedness	Functional fixedness is the tendency to conceptualize an object (broadly construed) only in terms of its most common use.	Clinicians may believe that the trauma narrative can only be done in the way that it has been taught to them. For example, if a clinician is only taught to implement the trauma narrative verbally, they may struggle to consider other methods/modalities by which to implement it.
Hopelessness/Helplessness	Hopelessness and helplessness are the feelings that things will not get better and that there are no ways to improve the situation.	Clinicians may feel “stuck” when attempting to implement the trauma narrative because several other barriers or challenges have intervened their ability to implement it. Clinicians may feel that despite their attempts to implement the trauma narrative, due to factors outside of their control (e.g., the client’s psychosocial stressors, their inability to attend sessions) they are being insufficiently rewarded for their work, and therefore may be less inclined to attempt it with some clients.
Lack of Reinforcement	The lack of reinforcement is the absence of a reward that can strengthen a response or action.	Clinicians may feel they are not being rewarded for the uncompensated work they have to do to prepare for the trauma narrative session.
Risk/Loss Aversion	Loss aversion refers to the idea that losses are more painful than similar gains. This leads people to avoid risks when losses are involved.	Clinicians may perceive the risk of harm in conducting the trauma narrative as more salient than the benefits it may offer.
Social Norms	Social norms represent a psychological phenomenon in which people do something primarily because other people like them are doing it.	Clinicians may feel that if others at their agency are/are not using the trauma narrative, then they will be less/more likely to use it.

*Note*. Behavioral insights are largely selected from the Behavioral Economics Guide 2018 [58] with several additions included by study coders with expertise in behavioral insights

## Results

### Demographic characteristics

Qualitative interview participants were women (*n =* 17, 100%), master’s level (*n* = 17, 100%), predominantly licensed clinicians (*n* = 11, 65%) with a mean age of 32.24 years (*SD* = 9.74). The racial makeup of the sample was predominantly White (*n =* 15, 88 %), with other participants identifying as Black or African-American (*n* = 1, 6%) and other (*n* = 1, 6%). The majority identified as non-Latinx (*n* = 13, 76%). See Table 2 for sample demographic characteristics.

**Table 2:** Demographic characteristics

Characteristic	N (%) or Mean (SD, range)
**Age**	32.24 (9.74, 21-62)
**Gender (Woman)**	17 (100%)
**Hispanic/Latinx**	
Yes	4 (23.53%)
**Race**	
American Indian/Alaskan Native	0
Asian	0
Black or African American	1 (5.88%)
Native Hawaiian or Pacific Islander	0
White	15 (88.24%)
Other	1 (5.88%)
**Licensed**	
Yes	11 (64.71%)
**Which License?**	
Professional Counselor	2 (11.76%)
Clinical Social Worker	5 (29.41%)
Licensed Social Worker	3 (17.65%)
Marriage and Family Therapy	1 (5.88%)
Not Licensed	6 (35.29%)
**Highest Degree?**	
MA	4 (23.53%)
MS	4 (23.53%)
MEd	1 (5.88%)
MSW	6 (35.29%)
DSW	1 (5.88%)
MSS	1 (5.88%)
**Profession**	
Social Worker	8 (47.06%)
Professional Counselor	9 (52.94%)
**Years Practicing**	6.00 (7.84, 0.83-31.17)
**Completion of PACTS Training**	
2011	1 (5.88%)
2015	3 (17.65%)
2016	2 (11.76%)
2017	4 (23.53%)
2018	6 (35.29%)
No Response	1 (5.88%)

*Note*. Many clinicians in public mental health settings work under the license of their supervisor (often a licensed social worker or licensed professional counselor)

Forty-one percent of participants reported using TNs with most or all of their TF-CBT clients in the past 6 months. Seventy-six percent of clinicians said it was “very likely” they would use TNs with their TF-CBT clients in the next 6 months.

### Major findings

Three broad themes emerged from our analyses of clinicians’ responses (see Additional file 2 for coding results and Table 3 for TN determinants, behavioral insights, and strategies).

**Table 3:** Results from the behavioral insights informed analysis of interview data

Broad theme	TN determinant	Evidence from interview	Behavioral insights	Potential implementation strategy
**Decision Complexity (i.e., dimensions of clinicians’ decisions)**	Decision complexity surrounding the incorporation of other evidence-based interventions	4: “I feel like sometimes I might get a little bit stuck in the structure part and have a little bit of a harder time figuring out how to be flexible.”	Functional Fixedness Mental Models	Distribute stories/guides from similar clinicians (or peers) describing how they incorporate EBP with existing therapy routines.
		8: “I’m getting trained in Theraplay which is an evidence-based play therapy practice and because of the age group I’m working with right now I feel like that’s very helpful … I also employ, obviously, a lot of art therapy techniques”		
	Decision complexity surrounding client characteristics	17: “I find older children tend to be easier to do trauma narratives with than younger kids. I have a 4-year-old right now and it’s been kind of a process to figure out how to adapt TF-CBT to do a narrative with them. And then I also work with a 6 year old who doesn’t read yet, so I definitely feel like it’s easier when a kid is more verbal, and is of age to read on his own.”	Base Rate Fallacy /Mental Models Choice Overload/ Decision fatigue	Show clinicians narratives of kids with challenging presenting symptoms, or who may seem ill-suited for the narrative initially.
		10: “[My client is] juvenile justice-involved, and they said like her IQ’s 76 and verbal comprehension is by far her lowest competency, so that’s helped us reframe our whole therapeutic approach. We’re just doing so much more attachment-oriented things with her mom, who’s also intellectually disabled. And so it’s like this kid needs it.”		
	Decision complexity surrounding client psychopathology and/or complexity of trauma.	10: “[Barriers:] Cases where there’s just like a lot of complex trauma. They were sexually abused, and they witnessed someone kill someone, and domestic violence. You’re like, how am I going to ever get to all of these things? Which ones are the things that are worth prioritizing?”	Base Rate Fallacy/Mental Models Choice Overload/ Decision Fatigue	Develop a decision aid (such as a checklist, trauma hierarchy, or flowsheet) which uses the client’s symptoms and other clinical characteristics to guide trauma narrative priorities.
		16: “I guess [I prioritize] just going based off of what they were sharing and prioritizing interventions to meet what they identified as bothering them the most.”		
		17: “I’ve had one kid who has had one singular trauma and it’s kind of been a process where we’ve really been able to follow TF-CBT to a T, it’s kind of progressing as expected and so forth.”		
**Clinician Affective Experience (i.e., emotions of the clinician)**	Clinician affective experience of the structure and/or flexibility of TF-CBT	14: I think maybe a template or something that can be given to clinicians to... you know, like “this chapter is about me” or, I don’t know, something to make it more user friendly. To feel like even though it’s unstructured, there’s some parameters around it. Interviewer: from your experience supervising, do you find that some therapists have more or less difficulty with the unstructured aspect of it? 14: Definitely. And I feel their own hesitation and worries about doing it creates … it takes longer to get there for their clients, and that’s not ok.	Risk Aversion Fear Avoidance	Develop a toolkit or workbook of resources for each module that makes it easy to be creative, while also being a template for those who are unsure as to how to implement the narrative.
		14: “I love the lack of structure, cause I’m creative”		
		14: “I know some of my staff that I supervise have [faced a lot of challenges]. Sometimes it’s more of this unstructured creativity part that they do not feel like they have the skill to do. One of the worries is where is it going to go, and if it goes somewhere we don’t want it to go, is that too much? We talked about the avoidance for me.”		
		11: “My supervisor is really helpful, my co-worker is really helpful. Also, I just go back the material, like a cheatsheet I keep with me.”		
	Clinician affective experience of client attendance	2: “Not only do gaps in attendance interrupt the therapeutic process, but some of my clients might have other behavioral issues, or other mental health issues, like poor recall, or they might be dealing with ADHD, so it’s almost like you’ve got to start over again”	Lack of Reinforcement Hopeless/ Helplessness	Incentivize clients to attend session with compensation and arrange transportation to bring the client to session.
		17: “It was really hard for clients and families to really be able to retain the information when there were gaps between the different skills we were learning as well as the gradual exposure that’s so paramount when you’re doing a narrative. And then it was also harder to see that hope of the more and more you do narrative and gradual expose the less distressed a kid is while hearing it and that was not really happening because there is too long of a period in between.”		
		13: “You explain the attendance contract. You say this is really important and the reasons why we’re doing trauma work. This is something that has to be built upon. You have to practice it. You’re coming every week to make sure the skills are being set, and if you’re not being consistent, then it is really hard to move forward.”		
	Clinician affective experience of the possibility of clients decompensating.	1. [Clinician reported that in the middle of the session she decided against doing the TN because the caregiver relapsed on substance abuse problems.] Interviewer: “What do you think would have happened if you had just gone forward with using the trauma narrative with that patient against your clinical gut?” Clinician: Probably a break in the relationship. Some transference would have probably . . . Yeah.	Risk Aversion	Use clinical supervision to do an imaginal exposure about a client decompensating.
		8: “That was something I really got supervision with my supervisor from and she was kind of supporting me in that and I felt like he was a kid that I could pull more from and really push whereas other kids again, I don’t know if I would be that comfortable doing that.”		
	Clinician affective experience of hearing the gory and difficult details of the trauma narrative.	14: “The murder part, the shooting that he witnessed was the goriest thing I’ve ever heard.”	Fear Avoidance/ Ostrich Effect	Develop a peer consultation model where clinicians can support one another and discuss challenging cases.
		5:“The intensity of asking the specifics, just the acuteness in the moment when someone’s telling a story, you know … This idea that we’re sharing a story; we pass around stories; we learn from this. There are so many examples of how narratives are so healing and art in the world.”		
	Clinician affective experience surrounding the clients’ social context and resource depravation	3: “They are constantly . . . they were displaced for a long time, and they are constantly about to be displaced. I feel like she is at a point right now, where it’s not . . . we could process the trauma with mom, but I don’t think that what’s happening for him substantiates doing TF-CBT.”	Lack of Reinforcement Helpless/ Hopelessness Mental Models	Assign case managers to provide support around basic needs so clinician can focus on clinical/therapeutic work
		10: “Look, we cannot get to this deeper work until Mom stops kicking the kid out of the house and locking the door.”		
		5: “But dad threatened him with a gun, so that shook up everything that we were doing … In those moments, I switch hats: I go into DV counselor mode and then immediately go into safety planning and really working hard with the parent to make sure we’re supporting whatever they need to do … We haven’t been able to get back on track with the components, and … we haven’t . . .been doing trauma narrative even though technically with the timing I should be doing that now.”		
		5: “And, you know, I’ve been meeting with him [my client] way longer than TF-CBT suggests. Really, since February, and I feel a lot of that is building that rapport … He, after seven months, was able to do the trauma narrative and talk about what happened, but it really did take that long, and I think sometimes the impression in the [TF-CBT supervision] call’s is that this is short-term, you know. Bam, bam, bam. You’re doing all the things, and they’re done and cured … This really showed me the amount of trust that you have to engender in your clients in order for it to be effective … I’m lucky because my agency does give me a lot of leeway … But just the mentality around, ‘we should be doing short-term intervention.’ Right?. . . every kid has a different time line		
**Agency Norms (i.e., social norms and practices at the workplace)**	Norms related to whether clinicians, supervisors, or agency leaders do or do not prioritize TF-CBT.	1:“We recently just . . . I think devoting time specifically to talking about TF-CBT is a barrier. I think the administrative piece takes precedent sometimes, and sometimes the clinical work or—I don’t want to say the quality of the clinical work but—the supervision of the clinical work, sometimes can get lost in the administrative piece.”	Social Norms Default Bias	Supervisors set an expectation that implementing the trauma narrative is a default by using templates in the Electronic Health Record, with a prompt for clinicians to make an accountable justification if they did not attempt the trauma narrative in session.
		8: “I guess if I were to go to another organization where TF-CBT was not so heavy, maybe I would stray away from it … maybe if I were to go into private practice, I don’t know how much I’d use them … yeah, if I changed jobs, or went to private practice something like that I might not do it to the extent that I am.”		
		8: “Where I work we do employ TF-CBT, that’s kind of what they do there. So I do a trauma narrative with every single kid.”		

#### Decision complexity

Decision complexity refers to the dimensions of a decision problem. The more dimensions of the problem, the more complex it is [64]. Behavioral insights suggest that more complex decisions lead clinicians to take longer to decide, to make more errors when they do, and to feel less confident in their decisions [65].

##### TN determinants

Clinicians who were overwhelmed by the complexity (e.g., client psychosocial and symptom complexity, client developmental level, and the variety of therapeutic techniques available) cited it as a major barrier to TN use. They described a high level of uncertainty once several features of their clients did not map onto their schema of a typical TF-CBT client. Conversely, other clinicians were able to reduce the complexity of decisions through processes like staging (i.e., breaking the decision up into its essential parts) or using decision aids [66]. Clinicians experienced in other EBIs described their skills as an asset, embracing the flexibility of the model.

##### Behavioral insights

The TN determinants revealed several behavioral insights: *choice overload/decision fatigue*, *base rate fallacy/mental models*, and *functional fixedness*. *Choice overload* is a cognitive process in which people have difficulty making a decision when faced with many options. This phenomenon is related to *decision fatigue*, which describes how the more decisions clinicians make, the poorer their clinical judgement [67]. When clinicians encounter clients with severe psychopathology, psychosocial stressors, and other challenges, they feel overloaded or fatigued. Other clinicians reported strategies such as accepting that TNs would not solve all of their clients’ problems, reframing their goals, or reducing their choices.

Clinicians who described that certain clients were better suited to creating TNs were potentially committing the *base rate fallacy* and revealed their specific *mental models*. The *base rate fallacy* arises when clinicians believe that aggregated data do not apply to individual clients. *Mental models* are people’s internal representations of a problem. Clinicians revealed that their vision of a “straight-forward” TF-CBT case is different from the cases they see. *Functional fixedness* captures clinicians’ perception that TNs can only be expressed in written form—the particular way they were trained to implement TNs. This prevents clinicians from integrating other clinical skills that would facilitate recovery. Clinicians who incorporated other techniques understood the purpose of the TN as a therapeutic tool beyond understanding how it is regularly implemented.

##### Implementation strategies

We used EAST to develop an implementation strategy that would disrupt clinicians’ *mental models* and *functional fixedness*. Showing clinicians that peers working in similar contexts can use techniques from other EBIs (e.g., evidence-based play therapy) may prompt clinicians to have more flexible *mental models* while at the same time providing a leading example for how other EBIs can be incorporated into the implementation of TNs [68, 69]. This would enable clinicians who are more flexible to influence those who are less flexible. This strategy would involve clinicians who incorporate other EBIs into TNs distributing stories or descriptive guides.

For clinicians who believe that certain client characteristics make TNs easier/harder, revealing *mental models* and *choice overload*, we generated a strategy in which supervisors show clinicians narratives of clients with challenging presenting symptoms, or who may seem ill-suited for TNs initially. This would provide a blueprint for clinicians with challenging clients. For clinicians who are concerned about their clients having their basic needs met, feeling *helpless*/*hopeless*, we designed a strategy to ease their burden. For clinicians with *choice overload/decision fatigue* relating to their clients’ severe psychopathology, we proposed a decision aid (such as a checklist, trauma hierarchy, or flowsheet) which uses the client’s clinical characteristics to guide TN priorities. Decision aids are behavioral insights-informed strategies for choice overload/decision fatigue [66].

#### Affective experience

Invariably, implementing psychological EBIs can provoke intense emotions. Trauma therapy is well known to cause clinicians distress. These emotions can, in turn, influence the quality of clinical decisions [70–72]. Evidence also suggests that clinicians working in high poverty contexts tend to experience additional stress given the enormous needs of their clients and the feeling of powerlessness this can engender [73].

##### TN determinants

Some clinicians described feeling overwhelmed by their clients’ economic hardships or by their clinical severity. Other clinicians described feeling distressed listening to graphic TNs or feeling afraid to push clients too far. Some indicated that the model is insufficiently concrete, leading them to feel anxious and uncertain. Many described not feeling rewarded for their uncompensated session planning and losing hope in clients’ improvement due to long treatment gaps or family disengagement. Contrary to clinicians who reported feeling overwhelmed by TNs (either due to their flexibility or their content), other clinicians reporting seeking guidance and support from their supervisors and reframing their perspective about TNs. Clinicians who might feel disappointed by inconsistent attendance instead created rules to ensure that clients would consistently attend.

##### Behavioral insights

These determinants revealed several behavioral insights: *risk/loss aversion*, *fear avoidance/ostrich effect*, *lack of reinforcement*, *helplessness/hopelessness*, *base rate fallacy/mental models*, and *functional fixedness*. *Risk/loss aversion* is the tendency to prefer avoiding losses to acquiring similar gains. Clinicians may perceive the risk of conducting TNs as more salient than the benefits they offer. *Fear avoidance* is the tendency to avoid thoughts or actions that cause people fear. The *ostrich effect* is a related phenomenon; it describes people’s tendency to ignore obvious, often negative, information because it is inconvenient or anxiety-inducing. Clinicians may avoid implementing TNs because they are difficult—clinicians may not be as skilled in TN delivery as they are in other practices. Clinicians may fear doing something that makes them feel incompetent. Some described dreading TN details because they are graphic and potentially produce vicarious traumatization.

Positive reinforcement describes the increased frequency of behaviors when they result in rewards [74]. Some clinicians described not feeling rewarded for their work, specifically for uncompensated TN preparation (e.g., session planning), as well as for their sustained attempts to help clients whose treatment was often derailed by more acute needs (e.g., psychosocial stressors). Despite clinicians’ attempts to implement TNs, due to factors outside of their control (such as clients’ crises that lead to missing sessions), they described feeling insufficiently rewarded—i.e., clients were not getting better. This *lack of reinforcement* may have led them to feel less inclined to attempt to implement TNs. Persistent *lack of reinforcement* led clinicians to experience helplessness and hopelessness about their clients’ progress and disappointment that TNs were not a panacea. Some clinicians avoided experiencing the *lack of reinforcement*, *helplessness*, and *hopelessness* by managing their expectations and reframing their goals.

Clinicians who described being able to manage their expectations and goals for clients viewed TNs as easier to implement, and displayed less *risk/loss aversion*, *fear avoidance*, and *helplessness/hopelessness*. They understood that they could not solve everything in their clients’ lives, which may have allowed them to reframe their expectations and mitigate the potential *lack of reinforcement*. Some clinicians reported seeking support and encouragement from their supervisors, reaffirming the rationale of TNs to themselves, and planning ahead to ensure that clients did not consistently lose momentum. Clinicians’ strategies to seek positive reinforcement from their supervisors/agencies enabled them to feel rewarded for their efforts irrespective of the forces outside of their control.

##### Implementation strategies

For clinicians who reported anxiety about the flexibility of the narrative, we generated an implementation strategy that would prevent this anxiety and provide concrete assistance to narrow the possibilities. We suggested the development of a toolkit or workbook of resources for TNs, serving as both a template and a toolkit of creative ideas. Some TF-CBT clinicians cited already using templates as helpful in alleviating their anxiety. Given that this anxiety appears to stem from an intolerance of uncertainty, providing concrete tools for clinicians can assuage their worries [75].

For clinicians who reported losing momentum due to clients’ inconsistent attendance, we developed a strategy that would reduce the frustration and worries of clinicians by incentivizing clients to attend session with financial compensation and arranged transportation. This would indirectly address the affective experience of clinicians by making it less likely that clients miss sessions. For clinicians who experience significant emotional distress about TNs (i.e., worrying that clients will decompensate or that the details will be difficult for them to hear), we generated implementation strategies to directly address clinicians’ anxieties through supportive techniques. One strategy involves using clinical supervision more therapeutically, acknowledging that clinicians also experience secondary traumatic stress. One technique that can be employed in group supervision is to do an imaginal exposure to feared outcomes (e.g., a client decompensating), effectively treating clinicians’ anxieties [76, 77]. We also generated a peer consultation model strategy where clinicians can support one another and discuss challenging cases. These practices would be incorporated into the supervision model (creating a default) which would reduce the effort of clinicians to seek support independently. The social element of the supervision and consultation models would make it more likely that clinicians feel supported and not alone. Assigning a case manager to provide support around clients’ basic needs would enable clinicians to focus on their therapeutic work and eliminate their worries that they should be prioritizing non-therapeutic casework. Equipped with the knowledge that their clients would be cared for, this strategy would help clinicians feel less *hopeless* about their clients’ prospects.

#### Agency norms

The final broad theme was agency norms—the social norms of clinicians’ agency leaders, supervisors, and peers. Evidence suggests that social norms strongly influence behavior [78].

##### TN determinants

Clinicians reported that if it was standard practice to use TNs in their agencies, clinicians would employ TNs. When agency leaders, supervisors, and colleagues did not prioritize TNs, clinicians reported that they were less likely to use TNs.

##### Behavioral insights

Agency norms reveal the behavioral insight that clinicians are influenced by the *default bias* and *social norms*. Clinicians prefer the current state of affairs, or the default practices they typically use in their clinical work. This *default* is taken as a reference point, and any change from that baseline is perceived as less preferable and sometimes aversive. *Social norms* arise when people do something primarily because others like them do. Clinicians are influenced by others at their agency who do or do not use TNs.

##### Implementation strategies

To address *social norms* and *default bias*, we generated an implementation strategy that makes use of the electronic health records clinicians typically use to record progress notes. Agencies and supervisors would create templates in the electronic health record that would require clinicians to describe their attempts to implement TNs. Clinicians would be prompted to write a justification if they did not attempt a TN in session with the knowledge that their supervisors would see the note. Establishing a *default* ensures that the standard practice is to use TNs, and, further, it creates a social norm that everyone at the agency implements TNs. Strategies prompting clinicians to provide justification embedded in electronic health records have been effective at increasing the use of other EBIs in medical settings [79].

## Discussion

Our study combines an in-depth qualitative analysis and a systematic application of theoretical principles from behavioral insights to understand implementation of an effective EBI for youth with PTSD [57]. We interviewed clinicians to identify implementation determinants of a core component of TF-CBT, the TN. We generated novel implementation strategies to target the hypothesized behavioral insights determining implementation behavior. The study identified three major themes relating to why clinicians do or do not use TNs: (1) decision complexity, (2) affective experience, and (3) agency norms. First, clinicians working in public mental health settings feel they are faced with particularly complex clients and contexts and have trouble translating clinical guidelines to practice. We generated implementation strategies that reduce decision complexity through decision aids and offloading responsibilities from clinicians. Second, the affective experience of clinicians implementing TNs in resource-scarce environments with severe clients leads them to feel overwhelmed and anxious. Clinicians’ experiences can be targeted through anxiety prevention strategies and therapeutic and emotionally supportive practices at the organizational level. Third, agency norms reflect clinician perceptions of what is considered standard practice in their agencies and determines TN use. The behavioral insights-informed strategy involves changing agency practices to facilitate TN implementation.

These results broaden our understanding of EBI implementation by analyzing clinicians’ lived experiences with theories on judgment and decision-making to design targeted and novel implementation strategies. Our study suggests that behavioral insights can provide a coherent theoretical guide across the implementation research continuum (from identifying determinants to practical guidance for implementation strategy design and selection) [80, 81]. This approach allowed us to go beyond the face-value understanding of clinicians’ first-person accounts and to develop hypotheses about the behavioral insights that may explain both clinicians’ behavior and clinicians’ understanding of their own implementation behavior.

One advantage of extending qualitative data beyond their immediate and literal meaning, particularly through the use of behavioral insights theories, can be demonstrated by example. Some clinicians in our study reported that their clients had more complex and frequent traumas than “most” clients—presumably than the typical child seeking trauma therapy. This barrier, if read literally, might be coded as “clinician knowledge and beliefs” under other widely used implementation frameworks [82]. Analyzing our data using behavioral insights offers us an additional lens through which to understand implementation behavior. We determined that this commonly voiced refrain may instead reveal clinicians’ choice overload [83]. When faced with an overwhelming amount of information, individual decision-makers tend to give up on their intended behaviors and offer post-hoc rationalizations for why they did not engage in them. Clinicians in public mental health settings likely encounter more severe and more complex clients than TF-CBT RCT participants—indeed, we have data to support this belief in Philadelphia [84]. However, little data support the notion that more severe clients would not benefit from the TN, though their symptoms may not fully remit [85]. The hypothesis that choice overload is the underlying psychological driver of clinicians’ behavior does not map onto an attitudinal implementation strategy; rather it maps onto designing a decision aid to distill a complex decision into a simpler, more digestible format. Decision aids are known to reduce complexity and simplify clinical decisions to optimize and improve clinical judgment [86]. Through using this behavioral insights-informed approach, we generated hypotheses about what drives implementation behavior based on what is latent in the qualitative data, but not literally stated by clinicians. This interpretative leap has its pitfalls—the hypothesized determinant may not apply—but we can justify our understanding through the extensive empirical literature that validate behavioral insights. In future research, the behavioral insights-informed implementation strategies can be evaluated for their effectiveness and our hypotheses about the behavioral insights serving as the mediating pathways can be tested.

Our study fits well within the literature on TF-CBT implementation. TF-CBT has been disseminated and implemented through various methods including remote web-based learning, live training, ongoing phone consultation, learning collaborative models, and some combination therein [87–90]. These efforts have been undertaken in a variety of public mental health settings in the USA and in low- and middle-income countries around the world [91–93]. Many of these studies’ results are consistent with our findings on clinicians’ self-reports of the challenges to TF-CBT implementation. For example, clinicians across settings believe that their contexts are quite different from the contexts of RCT trial participants. Our work also validates research from TF-CBT national trainers that TN adherence is low [17]. Thus, our work’s focus on clinicians’ perspectives of TNs, considered an active component of TF-CBT by treatment developers and national trainers, is an essential contribution to TF-CBT implementation research.

### Limitations

There were several limitations to our study. First, our analysis was based on clinician self-report. There is an inherent tension between attempting to discover the often unconscious psychological drivers of TN implementation and relying on clinicians’ self-report [94]. For example, data from the national trainers suggests that general psychotherapy competence is a core challenge for TF-CBT implementation [17]. Yet, none of the study participants described feeling incompetent, potentially confirming the behavioral insight that people prefer attributions that are self-enhancing over those that are self-deprecating [95]. Though qualitative data were analyzed beyond their immediate meaning, complementary quantitative measures of clinicians’ behavior (e.g., effectiveness and skill) were not collected that would have provided more data to understand implementation determinants.

Second, our approach primarily addresses individual clinicians’ decisions. Though we generated implementation strategies that target organizations (e.g., developing peer consultation models, transforming the electronic health record, hiring case managers, etc.), behavioral insights are less well suited to address organizational challenges or structural barriers (e.g., scarcity of resources) of which there are many in publicly funded mental health systems [7, 96]. Studying mental healthcare delivery calls for attention not just to individual clinician decisions, but to a structural understanding that takes all levels of analysis into account [1, 97]. Public mental health agencies in Philadelphia have benefited from a fertile policymaking ecology, which has incentivized EBI implementation. How policy decisions interact with outer context and individual clinician decision determinants was not explicitly explored, though clinicians often described these complex interactions. Ultimately, all solutions to serious public mental health concerns involve transforming individual behavior as the final link in the chain, making the analysis of judgment and decision-making critical. Future work should uncover both the potential and the limits inherent to examining individual clinician decisions as the unit of analysis to understand EBI implementation.

## Conclusions

In-depth qualitative interviews revealed that clinicians implementing TNs—an active component of an effective EBI, TF-CBT—in public mental health agencies are faced with challenges relating to decision complexity, their affective experiences, and agency norms. We generated behavioral insights informed by hypotheses about what determines clinicians’ implementation behavior and designed corresponding implementation strategies using an established behavioral insights framework (EAST). Future research will test these implementation strategies to understand if and how they work. This work will also integrate the behavioral insights discovered from this approach with insights from implementation science frameworks that account for structural and organizational contexts. The goal of our work is to synthesize interdisciplinary knowledge to determine the factors that impede and facilitate EBI implementation, and to test methods to improve implementation. Understanding clinical judgment and decision-making will enhance our capacity to design effective approaches to improve healthcare.

## Supplementary Information

**Additional file 1.** Qualitative Interview Guide.

**Additional file 2.** Behavioral Insights Coding Process and Results.

## Data Availability

The dataset generated and analyzed during the current study is not publicly available due to the highly sensitive nature of interview transcript data. Publication of entire transcripts risk identifying research participants.

## Abbreviations

DBHIDS: Department of Behavioral Health and Intellectual Disability Services
EAST: Easy Attractive Social and Timely
EBI: Evidence-based Intervention
NUDGE: Narrow, Understand, Discover, Generate, and Evaluate framework
PACTS: Philadelphia Alliance for Child Trauma Services
TF-CBT: Trauma-Focused Cognitive Behavioral Therapy
TN: Trauma Narrative
