# Correction to: Using behavioral insights to design implementation strategies in public mental health settings: a qualitative study of clinical decision-making

**DOI:** 10.1186/s43058-021-00115-y

**Published:** 2021-02-01

**Authors:** Briana S. Last, Simone H. Schriger, Carter E. Timon, Hannah E. Frank, Alison M. Buttenheim, Brittany N. Rudd, Sara Fernandez-Marcote, Carrie Comeau, Sosunmolu Shoyinka, Rinad S. Beidas

**Affiliations:** 1 Department of Psychology, University of Pennsylvania, Philadelphia, PA USA; 2 College of Liberal and Professional Studies, University of Pennsylvania, Philadelphia, PA USA; 3 Department of Psychology, Temple University, Philadelphia, PA USA; 4 Department of Psychiatry and Human Behavior, Warren Alpert Medical School of Brown University, Providence, RI USA; 5 Department of Family and Community Health, School of Nursing, University of Pennsylvania, Philadelphia, PA USA; 6 Center for Health Incentives and Behavioral Economics (CHIBE), University of Pennsylvania, Philadelphia, PA USA; 7 Penn Implementation Science Center at the Leonard Davis Institute of Health Economics (PISCE@LDI), University of Pennsylvania, Philadelphia, PA USA; 8 Department of Psychiatry, University of Pennsylvania Perelman School of Medicine, Philadelphia, PA USA; 9 Department of Psychiatry, University of Illinois at Chicago, Chicago, IL USA; 10 Community Behavioral Health, Philadelphia, PA USA; 11 Department of Behavioral Health and Intellectual Disability Services, Philadelphia, PA USA; 12 Department of Medical Ethics and Health Policy, University of Pennsylvania Perelman School of Medicine, Philadelphia, PA USA; 13 Department of Medicine, University of Pennsylvania Perelman School of Medicine, Philadelphia, PA USA

**Correction to: Implement Sci Commun (2021) 2:6**

**https://doi.org/10.1186/s43058-020-00105-6**

Following publication of the original article [1], the authors noted that Rinad S. Beidas was missing affiliation 8: Department of Psychiatry, University of Pennsylvania Perelman School of Medicine, Philadelphia, PA, USA.

This affiliation has been added to Rinad S. Beidas in the author list and the original article [1] has been corrected.
