# Supplementary material for: Using behavioral insights to design implementation strategies in public mental health settings: a qualitative study of clinical decision-making
Source: Implement Sci Commun. 2021 Jan 11;2:6. doi: 10.1186/s43058-020-00105-6 (PMC7802291; doi:10.1186/s43058-020-00105-6)
Supplement: Supplementary file 1 — Additional file 1. Qualitative Interview Guide. [file 43058_2020_105_MOESM1_ESM.docx]

**Appendix A**

Qualitative Interview Guide

**General Introduction Script:**

Thank you for agreeing to participate. My name is [**Interviewer’s Name]**, and I am affiliated with the University of Pennsylvania.

As part of PACTS, you have been trained to use trauma-focused cognitive behavioral therapy (TF-CBT) to treat children with a history of trauma. TF-CBT includes several elements, but today we are specifically interested in hearing your thoughts about using the trauma narrative. A trauma narrative is defined as the method through which the clinician encourages the youth to share the details of events associated with the traumatic experiences, including thoughts, feelings, and sensations experienced at the time of the trauma. The trauma narrative typically involves the writing or dictating/telling of a narrative, but may take other creative forms (e.g., poetry, songs, plays or art).

We know that regularly using trauma narratives can sometimes be difficult. There are probably advantages and disadvantages to using trauma narratives. In a previous survey, you indicated some of the advantages and disadvantages of using trauma narratives when delivering TF-CBT. Today I will ask about your experience and perceptions of using trauma narratives generally, using your responses on the previous survey to guide our discussion. I have prepared some questions that will take no more than one hour to answer. You can spend as much or as little time as you like answering each question. At the end of the interview, you will receive $50 for your participation.

Our goal is learn more about your perspective on using trauma narratives as a part of TF-CBT. It’s important for you to know that there are NO right or wrong answers. We are interested only in *your* opinions and perceptions.

Do you have any questions?

1. Tell me about your experience using trauma narratives when treating youth with a history of trauma.

*[Probes*:

- *Have you ever used them? [always ask if they did not respond to the first question with an answer about their personal experience using trauma narratives]*
- *How do you decide when or with whom to use them? Or when not to use them?*
- *Are there elements of trauma narratives that you particularly like or dislike?]*

1. What are some factors that make it hard to use trauma narratives?

*[Probes (If they do not generate responses on their own)*:

- *Are there any client characteristics that make it particularly hard?*
- *Anything about your organization that makes it hard?*
- *Anything about your supervisor?*
- *Anything about the amount of time you have to prepare?*
- *Is there anything that makes it hard for you personally/emotionally?*
- *Is there anything related to the procedures of completing trauma narratives that makes it difficult to use them?]*

1. What are some factors that make it easier to use trauma narratives?

*[Probes (If they do not generate responses on their own)*:

- *Are there any client characteristics that make it easier?*
- *Anything about your organization?*
- *Anything about your supervisor?*
- *Is there anything that makes it easier for you personally/emotionally?*
- *Is there anything related to the procedures of completing trauma narratives that makes it easier to use them?]*

1. Over time, things could make you change your mind about whether you intend to regularly use trauma narratives or not. *[For the following questions, use the same probes as in questions 2 and 3 if needed.]*
   1. Over time, what could make you lose interest in using trauma narratives? *[Why?]*
   2. What would make it more likely for you to use trauma narratives in the future? *[Why?]*
2. Tell me about a recent TF-CBT case when you used a trauma narrative(s). *Note: If they say they have never used a trauma narrative, proceed to question 6.*

*[Probes:*

- *What made you decide to use a trauma narrative with this particular child?*
- *What made it easier or harder to use a trauma narrative in this case?*
- *How did it go?*
- *How did you tailor the trauma narrative to this particular client?*
- *How did you decide when the child was ready to start working on the trauma narrative in session?*
- *How did you decide how many details to get from the child during the trauma narrative?*
- *How did you feel right before doing the trauma narrative? Right after?]*

1. Tell me about a recent TF-CBT case when you planned to use a trauma narrative, but did not implement it in session.

*[Probes*: {*Chain analysis of TN use}*

- What made you decide that you wanted to use a trauma narrative in this session?
- What happened during the session that made you decide not to use a trauma narrative?
- How did you feel/what were you thinking right before you planned to use the trauma narrative?
- How did you feel the remainder of the session after you decided not to do the trauma narrative?
- *Is this a typical TF-CBT case for you? If not, walk me through another case.*
- *What do you think would have happened if you had used a trauma narrative with this child?]*

1. *Provide list of facilitators for use of trauma narratives based on this participant’s responses to the initial survey*: Tell me more about how these factors apply to your use of trauma narratives.
2. *Provide list of barriers for use of trauma narratives based on this participant’s responses to the initial survey*: Tell me more about how these factors make it difficult for you to use trauma narratives.
3. Is there any additional information that you would like to share about your perceptions about or experience using trauma narratives?

**INTERVIEWER COMMENTS**

1. Respondent’s level of interest and involvement in answering questions.

| 1.  Very low | 2.  Low | 3.  Neutral | 4.  High | 5.  Very high |
| --- | --- | --- | --- | --- |

1. Please estimate the respondent’s understanding of the interview.

| 1.  Limited | 2.  Partial | 3.  Average | 4.  Majority | 5.  Complete |
| --- | --- | --- | --- | --- |

1. Please rate your impression of the knowledge of the respondent in the topic being addressed in this module.

| 1.  Highly Questionable | 2.  Somewhat Questionable | 3.  Neither | 4.  Somewhat Knowledgeable | 5.  Highly Knowledgeable |
| --- | --- | --- | --- | --- |

1. Describe any discrepancies, gaps, or other problems with the interview.
2. Describe any circumstances that occurred while the interview was in progress that may have affected the quality of the interview (i.e., interruptions)?
3. Describe any affective and/or non-verbal responses displayed by the participant:
