# Supplementary material for: Using behavioral insights to design implementation strategies in public mental health settings: a qualitative study of clinical decision-making
Source: Implement Sci Commun. 2021 Jan 11;2:6. doi: 10.1186/s43058-020-00105-6 (PMC7802291; doi:10.1186/s43058-020-00105-6)
Supplement: Supplementary file 2 — Additional file 2. Behavioral Insights Coding Process and Results. [file 43058_2020_105_MOESM2_ESM.docx]

**Appendix B**

**Behavioral Insights Coding Process and Results**

**Methods**

Figure 1 displays the multi-step process we used to analyze the qualitative interviews. In Step 1, three investigators (BSL, CET, and SHS) separately reviewed all of the qualitative interview transcripts. Investigators met and coded the determinants of TN use. Codes largely reflected clinicians’ responses to questions about the barriers (i.e., what prevents) and facilitators (i.e., what helps) TN implementation (see Appendix A for interview questions), though other TN determinants were inferred. For example, some codes were “complex trauma,” “community violence,” “back-to-back sessions.” In Step 2, using a thematic analytic approach, these determinants were organized into themes (e.g., “client characteristics,” “clinician affective experience,” “agency norms,” etc.). In Step 3, as a validation check, investigators coded four transcripts together and synthesized and deduplicated further. Through discussion and consensus, coders distilled and reduced the codes and broad themes.

In Step 4, three investigators (BSL, CET, and SHS) mapped the TN determinants, coded in the clinicians’ own language, onto a predetermined set of behavioral insights that are described in the Behavioral Economics Guide 2018 (58). Table 1 provides definitions of the behavioral insights that we mapped onto the TN determinants (see the guide for the full list). There were several cases in which well-established behavioral insights were not listed in the guide. For example, “reinforcement” is a psychological principle (97) that is not explicitly in the guide (though related insights such as “incentives” are). When these occasions arose, coders discussed whether it was appropriate to include these behavioral insights in our coding process, which resulted in several additions. We consider the few additions to be behavioral insights insofar as they derive from the behavioral science literature, have been shown to determine behavior, and can be shaped through behavioral insights strategies. In this step, it was possible for several behavioral insights to map onto one TN determinant, which explains why several behavioral insights are associated with one TN code (see Table 3). For example, “social norms” and “defaults” map onto the TN determinant code relating to common practices at a particular agency.

In Step 5, to ensure the validity of the TN determinants and behavioral insights generated from Steps 1-4, we conducted an expert validation check. BSL (a graduate student with clinical experience conducting TF-CBT and research expertise in clinical decision-making) worked with AMB (an expert in behavioral insights) and RSB (a licensed clinical psychologist and expert in implementation science) to validate the TN determinants and behavioral insights based on the literature and their research and clinical expertise. After this final list of hypotheses was validated, for Step 6, BSL and RSB integrated the behavioral insights and implementation science literature to generate implementation strategies, using the EAST framework to structure this process.

**Coding Results**

Step 1 generated 53 TN determinants, coded in the clinicians’ own language. In Step 2, we organized these 53 TN determinants into 11 broad themes using a thematic analysis. In Step 3, coders further reduced, distilled, and synthesized the TN determinants into 36 TN determinants under the 11 broad themes. In Step 4, the codes were mapped onto behavioral insights, and resulted in 18 behavioral insights organized under three broad themes, which the experts validated in Step 5. In Step 6, coders mapped the 18 behavioral insights (organized under three broad themes) onto 9 EAST-informed implementation strategies.

Table 3 displays the final list of three broad themes that organize the final set of TN determinants; the behavioral insights that correspond to them; and behavioral insights informed implementation strategies generated from this process. Exemplar quotes from clinicians are also included in the table to demonstrate the data we used to generate the TN determinants, behavioral insights, and implementation strategies.

It is important to note that the final broad themes, though sufficiently separable, are not entirely distinct. For example, many clinicians described the cognitive burden of being faced with too many decisions, but this choice overload was not entirely separable from the affective experience of being overwhelmed and anxious, nor was it entirely distinct from the agency norms that provided additional support surrounding TNs. These conceptual categories are abstractions to organize the data and cannot reflect the complex overdetermination of implementation behavior. Our analysis shows that the behavioral insights, due to their scientific validity, may offer a closer approximation of the underlying mechanism determining behavior.

***Decision Complexity***

**Overview of Coding Results.** Step 1 generated several TN determinant codes such as “complex trauma,” “cognitive level of client,” and “incorporating other therapies.” In Step 2, these codes were organized into broad themes such as “patient complexity,” “social context,” and “therapist flexibility/creativity.” In Step 3, these codes were further distilled and synthesized. In Step 4, these codes were organized into three broad themes, including decision complexity, and mapped onto several behavioral insights using the Behavioral Economics Guide. In Step 5, behavioral insights experts provided a check to validate the analysis of broad themes, TN determinants, and hypothesized behavioral insights. In Step 6, BSL and RSB generated implementation strategies leveraging clinicians’ self-reported strategies and using EAST, the behavioral insights informed framework.

***Clinicians Affective Experience***

**Overview of Coding Results**. Step 1 generated several TN determinant codes such as “roadmap,” “hopelessness,” “fear of decompensation, “I lose momentum,” and “gory details.” In Step 2, these codes were organized into broad themes such as “clinician confidence,” “public health context,” and “clinician affective experience.” In Step 3, these codes were further reduced and deduplicated. In Step 4, these codes were organized into three broad themes, including clinician affective experience, and mapped onto behavioral insights. In Step 5, behavioral insights experts provided an expert check on the broad themes, TN determinants, and hypothesized behavioral insights. In Step 6, coders generated implementation strategies leveraging clinicians’ self-reported strategies and using EAST.

***Agency Norms***

**Overview of Coding Results**. Step 1 generated several TN determinant codes such as “supervisor support,” “agency support” and “just what we do.” In Step 2, these codes were organized into the broad theme of “agency norms.” In Step 3, these codes were further reduced to one code relating to agency norms, which became a broad theme in Step 4. In Step 4, the code “agency norms” was mapped onto behavioral insights, with expert validation in Step 5, and strategy design in Step 6 using EAST.
